# A Pilot Study on the Concentration, Distribution and Bioaccumulation of Polybrominated Diphenyl Ethers (PBDEs) in Tissues and Organs of Grassland Sheep

**DOI:** 10.3390/ijerph191912170

**Published:** 2022-09-26

**Authors:** Wenming Chen, Xinrui Yang, Junsong Bao, Ziyi Lin, Tianwei Li, Ying Wang, Aiqin Zhang, Jicheng Hu, Jun Jin

**Affiliations:** 1 College of Life and Environmental Sciences, Minzu University of China, Beijing 100081, China; 2 Beijing Food and Environmental Health Engineering Center, Beijing 100081, China

**Keywords:** polybrominated diphenyl ethers, tissue distribution, ovine serum albumin, cytochrome P450 enzyme, molecular docking, molecular properties

## Abstract

Polybrominated diphenyl ether (PBDE) concentrations in various tissues and organs of grassland sheep from Inner Mongolia, China, were determined. The abilities of PBDEs binding to ovine serum albumin (OSA) and Cytochrome P450 enzyme (CYP3A24) were assessed by fluorescence spectroscopy and molecular docking simulations. The PBDE concentrations in the sheep tissue and organ samples were 33.4–167 pg/g dw. The distribution of PBDEs in sheep organs and tissues is affected not only by the function of organs and tissues, but also by the characteristics of PBDEs. Adipose tissue tends to bioaccumulate more-brominated BDEs (BDE-154, -153, and -183), but muscle tissues and visceral organs mainly bioaccumulate less-brominated BDEs. The distribution of PBDEs in visceral organs is mainly affected by the transport of ovine serum albumin (OSA) and the metabolism of CYP3A24 enzyme. The distribution of PBDEs in adipose tissue and brain is mainly affected by their logK_OW_.

## 1. Introduction

Polybrominated diphenyl ethers (PBDEs) are used as brominated flame retardants. PBDEs are widely used in coatings, electronics, furniture, plastics, and textiles. PBDEs have mostly been released into the environment from electronic products and electrical equipment [1], sewage treatment plants, and landfill sites [2]. Large amounts of PBDEs are released into the environment when products containing PBDEs are used and recycled [3]. PBDEs are lipophilic, persistent, bioaccumulative, and toxic. PBDEs can be transported over long distances in the environment and can reach remote alpine ecosystems [4].

Zhang et al. believe that the liver of birds living in the e-waste disassembly area was the main bioaccumulation organ of PBDEs because of the high concentration of PBDEs in the liver [5]. PBDEs concentrations in various tissues of chickens, ducks, and mice living near an e-waste disassembly site decreased in the order of liver > muscle > brain [6]. However, the livers of kestrels from Beijing and various raptors from Belgium were considered to be the main metabolic organs of PBDEs because the concentrations of PBDEs in these livers were low [7,8]. Staskal et al. orally administered BDE-47 to mice and found that the concentration of BDE-47 was the highest in adipose tissue, the levels of skin, liver, muscle, and lung were medium, and the levels of BDE-47 in kidney and brain were very low [9]. Sanders et al. also believe that BDE-47 was mainly contained in adipose tissue in rodents [10]. Most studies of PBDE distributions in animal tissues have been focused on PBDE concentrations in the liver and muscle tissue. Few studies of PBDE distributions among other tissues have been published. The distribution of PBDEs between different tissues and organs in an organism is affected by complex factors, and the distribution mechanism is not clear. Sheep are important domestic animals and can act as sentinels for environmental pollution [11]. So, studying the distribution of PBDEs in sheep tissues and organs is helpful to better understand the bioaccumulation mechanism of PBDEs.

Serum albumin is the most abundant protein in plasma and the main component of pollutant transport in blood [12]. After entering the body of an animal, pollutants form complexes with serum albumin and then are transported to various organs. It is important to explore the interaction between pollutants and serum albumin for understanding the migration mechanism of pollutants. Cytochrome P450 enzyme system (CYPs) is an important enzyme system involved in detoxification of exogenous compounds and metabolism of endogenous compounds [13]. CYP3A24 is considered to be the most important Cytochrome P450 enzyme isoform in sheep [14,15]. Therefore, the study of the combination between CYP3A24 enzyme and PBDEs can well explain the metabolism of PBDEs in sheep.

Fluorescence detection is useful for studying interactions between biological macromolecules and small molecule compounds [16,17,18]. Upon binding to small molecule compounds, endogenous fluorescent chromophores of proteins are able to undergo fluorescence quenching [16]. The intensity of the emission peak of serum albumin decreased regularly and gradually with the gradual increase of the concentration of small molecule substances, indicating that the interaction between small molecule compounds and serum albumin occurred [19,20,21]. Molecular docking can intuitively show the process and results of the interaction between molecules and proteins [22,23,24]. Shang et al. determined the binding constant (K_A_) and binding site number (*n*) of hesperidin and glycoside hesperidin with bovine serum albumin (BSA) by fluorescence spectrometry [25]. Tan et al. simulated the interaction between PBDEs and human serum albumin (HSA) by molecular docking [26]. Cao et al. used molecular docking simulation to study the degradation ability of biphenyl dioxygenase from *Enterobacter* sp. LY402 to PCBs [27]. Research showed that the binding energy of molecular docking had a significant correlation with the degradation rate constant of the enzyme, that is, the smaller the binding energy means the higher the degradation rate of the substrate by the enzyme. Therefore, the distribution mechanism of PBDEs in sheep tissues and organs can be explained by detecting the PBDE concentrations in sheep tissues and organs and determining the binding constant of the interaction between PBDE congeners and serum albumin and CYP3A24. This can improve our understanding of the absorption, migration, accumulation, and excretion of PBDEs in organisms.

## 2. Materials and Methods

### 2.1. Sampling

Samples were collected from the Xilingol grassland (116.36° E, 44.27° N, 1140 m above sea level) in Inner Mongolia, China, in July 2018. Xilingol Grassland covers an area of 179,600 square kilometers, far from the city, mainly focusing on animal husbandry. Two 1-year-old sheep were slaughtered (1-year-old sheep were sold by herdsmen). Then, samples of the abdominal fat (*n* = 1), tail fat (*n* = 1), chest muscle (*n* = 1), hind leg muscle (*n* = 2), brain (*n* = 1), heart (*n* = 1), kidneys (*n* = 1), liver (*n* = 2), spleen (*n* = 1), lung (*n* = 1), small intestine (*n* = 1), and stomach (*n* = 1) from a whole sheep were collected. Samples of tail fat (*n* = 1) and hind leg muscle (*n* = 1) from a sheep of the same breed raised by another herdsman were also collected. According to field investigation, sheep in Xilingol Grassland only feed on herbage. Samples of air (*n* = 3), herbage (*n* = 15), soil (0–10 cm deep, *n* = 2), and water (*n* = 5) were collected. The air samples were collected using polyurethane foam disks using a previously described method [28]. The samples were stored at −20 °C until they were extracted.

### 2.2. Chemical Analysis

The reagents and sample pretreatment methods that were used were described in previous publications [28,29]. Briefly, ^13^C_12_-labeled bromodiphenyl ether BDE-139 (2.5 ng) and BDE-209 (25 ng) were added to a 200 mL water sample to act as an internal standard; then, the sample was liquid–liquid extracted and the extract was evaporated to 5 mL. Each animal tissue sample was rinsed with ultrapure water to remove surface blood. Each non-water sample (soil, herbage, sheep tissue or organ) was freeze-dried, ground, and homogenized, and then, a 3.0 g sample was spiked with the internal standard (BDE-139 (2.5 ng) and BDE-209 (25 ng)) and Soxhlet extracted with 200 mL of a 1:1 *v*/*v* mixture of *n*-hexane and acetone for 24 h. The extract was cleaned by passing it through a composite silica gel column and then evaporated almost to dryness under a stream of nitrogen before 100 μL of nonane were added. The sample extracts were analyzed using an Agilent 6890 gas chromatograph coupled to an Agilent 5975 N mass spectrometer (Agilent Technologies, Santa Clara, CA, USA). The instrument settings were described in a previous publication [28,29]. Briefly, for PBDE congeners (excluding BDE-209), we selected DB-5MS column (30 m long, 0.25 mm i.d., 0.1 µm film thickness; J & W Scientific; Agilent Technologies) for separation. For BDE-209, DB-5 MS column (15 m long, 0.25 mm i.d., 0.1 µm film thickness; J & W Scientific; Agilent Technologies) was selected for separation. The starting temperature of the procedure was 100 °C, held for 3 min, then increased to 300 °C at 4 °C/min, and finally held at 300 °C for 30 min. The injector, quadrupole, and ion source temperatures were 320 °C, 290 °C and 150 °C, respectively.

A 2 mL aliquot of 1.0 × 10^−6^ mol/L ovine serum albumin (OSA) solution and 2 mL of a PBDE solution at a concentration of 0, 2 × 10^−7^, 4 × 10^−7^, 6 × 10^−7^, 8 × 10^−7^, or 10 × 10^−7^ mol/L were added to a 10 mL glass test tube. The solutions were mixed well and allowed to react at room temperature for 5 min. The mixture was then transferred to a quartz cuvette and a fluorescence spectrum was acquired using an F4500 fluorescence spectrometer (Hitachi High-Technologies, Tokyo, Japan). The excitation wavelength was 280 nm, the excitation and emission passbands were both 5 nm, and the fluorescence spectrum scanning range was 300–450 nm. For interactions between PBDEs and biological macromolecules such as OSA, the binding constant K_A_ and the number of binding sites *n* are related using the Lineweaver–Burk equation [30]

$$log\frac{F_{0}-F}{F}=nl{ogK}_{A}+nlog[(D_{t})-\frac{\left(F_{0}-F)(P_{t}\right)}{F_{0}}],$$

where *D_t_* is the PBDE concentration, *P_t_* is the protein concentration, *F*_0_ is the OSA fluorescence intensity without PBDEs present, and *F* is the OSA fluorescence intensity with PBDEs present. On a plot of log(*F_0_* − *F*)/*F* against log(*D_t_* − (*F*_0_ − *F*)*P_t_*/*F*_0_), log*K_A_* is the intercept and *n* is the slope, meaning the number of binding sites *n* and the binding constant *K_A_* can be determined.

Autodocktools 1.5.6 molecular docking software was used to simulate molecular docking of PBDEs with OSA. The OSA crystal structure (PDB ID 6HN0) was taken from the RCSB protein databank (https://www.rcsb.org/structure/6HN0, accessed on 9 October 2019) and the CYP3A24 crystal structure (ID Q29496) was taken from the UniProtKB (https://www.uniprot.org/uniprotkb/Q29496/entry, accessed on 2 December 2020). The structures of PBDEs were taken from a small molecule database (https://pubchem.ncbi.nlm.nih.gov/, accessed on 8 August 2005). The parameter settings of molecular docking referred to published research, that is, the grid box consisted of 126 × 126 × 126 points to provide enough space for molecular docking, where the ligand-centered map of OSA was with 0.375 Å [26]. A semi-flexible docking mode was used. Each PBDE was docked with OSA or CYP3A24 randomly 100 times. The Lamarckian genetic algorithm was used to optimize the energy, and the conformation with the highest score weight was selected as the final docking result. The binding free energy ΔG_binding_ was used to evaluate the effects of the PBDEs on OSA and CYP3A24. PyMOL visualization software was used to indicate molecular interactions and binding sites. Chemoffice software was used to calculate the steric resistance energies of the PBDE congeners.

### 2.3. Quality Control and Assurance

Each target compound was quantified using an internal standard method using the isotope-labeled internal standard and a five-point calibration. The correlation coefficient for each calibration curve was required to be >0.9990. The limit of detection (LOD) was defined as the concentration giving a signal-to-noise ratio of 3, and the limit of quantification (LOQ) was defined as the concentration giving a signal-to-noise ratio of 10. The LOQ ranged from 0.0033 (BDE-99) to 0.0067 (BDE-183) ng/g dw. The internal standard recoveries for the samples were 79.6–122%. No target compounds were detected in the blank samples. The relative standard deviations for PBDE concentrations found in two liver and two hind leg muscle parallel samples from the same sheep were 2.50% and 2.68%, respectively. The relative standard deviations for PBDE concentrations found in three hind leg meat samples and two tail fat samples from different sheep were 3.81% and 1.51%, respectively. The variation of parallel samples between different sheep tissues was small, which indicates that the PBDE concentrations in the collected sheep tissues are in a stable state.

### 2.4. Statistical Analyses

All data analyses were performed using GraphPad Prism version 5 and Origin version 18.0 software. The data were rounded to three significant figures. Each concentration below the quantitation limit was replaced with half of the quantitation limit before statistical tests were performed.

## 3. Results and Discussion

### 3.1. PBDE Concentrations in Grassland Sheep Organs and Tissues

**Figure 1:**
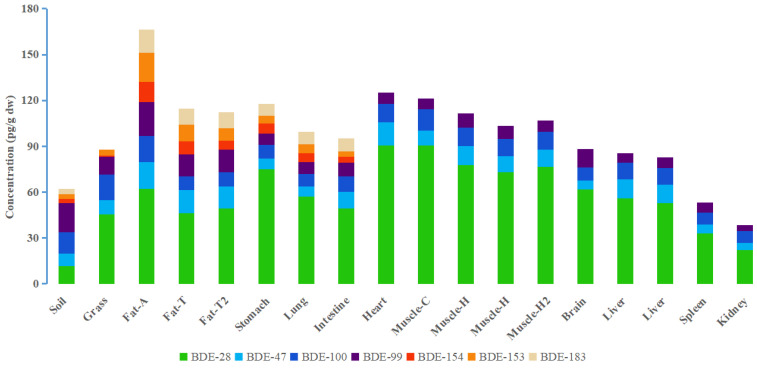
Polybrominated diphenyl ether concentrations in different tissues or organs from a sheep raised in a grassland area. Notes: Fat-A = abdominal fat, Fat-T = tail fat, Fat-T2 = tail fat of the second sheep, Muscle-C = chest muscle, Muscle-H = hind leg muscle, Muscle-H2 = hind leg muscle of the second sheep.

It has been found in many studies [8,31] that organic pollutant distributions in animal tissues and organs do not fully fit the “lipid compartment” distribution theory proposed by Matthews and Dedrick [32], i.e., the organic pollutant concentrations in various tissues and organs are not only related to the fat contents of the tissues and organs. Therefore, we used PBDE concentrations in the sheep tissue and organs on a dry weight (dw) basis to attempt to improve our understanding of how sheep absorb PBDEs and how PBDEs are distributed to different tissues in sheep. The PBDE concentrations are shown in Figure 1. BDE-209 was not detected in environmental samples and sheep tissue and organ samples in Xilingol grassland. The concentrations of PBDEs in air, water, soil, and grass in the sheep living area were 0.441 pg/m^3^, 3.67 ng/L, 61.9 pg/g dw and 87.6 pg/g dw, respectively. The sums of the BDE-28, -47, -99, -100, -153, -154, and -183 concentrations in the sheep tissue and organ samples were 33.4–167 pg/g dw (1.68 × 10^2^–1.84 × 10^3^ pg/g lw (lipid weight)). Nomiyama et al. found sums of the BDE-28, -47, -99, -100, -153, -154, and -183 concentrations in organs from stray dogs and cats in Japan were 12.51 ng/g lipid weight (dog liver), 0.17 ng/g lw (dog brain), 29.2 ng/g lw (cat liver), and 0.29 ng/g lw (cat brain) [33]. Li et al. found sums of the BDE-28, -47, -99, -100, -153, -154, and -183 concentrations in adipose tissue from pigs kept near an e-waste disassembly site in southern China were 20.2–28.9 ng/g lw [34]. The PBDE concentrations in the grassland sheep tissue and organ samples were lower than PBDE concentrations found in other animal tissues and organs because of the low background PBDE concentrations in the grassland area.

The ƩPBDE concentrations in the sheep organs decreased in the following order: heart (125 pg/g dw) > stomach (118 pg/g dw) > lung (99.5 pg/g dw) > small intestine (95.1 pg/g dw) > brain (88.3 pg/g dw) > liver (84.0 pg/g dw) > spleen (53.1 pg/g dw) > kidney (38.4 pg/g dw). In all organs, the highest concentration of PBDEs appears in the heart, which may be due to the high degree of blood perfusion through the heart, resulting in the high bioaccumulation of PBDEs. The stomach can absorb PBDEs from ingested grass and soil. The lung can be exposed to PBDEs via inhaled soil particles and air. It is not surprising that they have higher PBDE concentrations. The PBDE concentration in the small intestine sample was lower than the concentration in the stomach sample. The intestine is rich in anaerobic microorganisms, which can metabolize PBDEs [35]. The blood–brain barrier can hinder PBDEs accumulation in the brain. The liver can metabolize PBDEs [8]. The spleen filters the blood and can remove waste products from the blood. The PBDE concentrations in the brain, liver, and spleen samples were between the intake organ (stomach) and excretion organ (kidney). Among all tissues and organs, the level of PBDEs in kidney was the lowest. Since PBDEs have high logK_OW_ values, it may mean that some PBDE congeners were not easy to excrete through urine. PBDEs showed a high level in adipose and muscle tissue. The PBDE concentrations in muscle were high, possibly because the high degree of blood perfusion through muscle caused PBDEs strongly enriched. Adipose tissue has less blood perfusion than muscle tissue. However, PBDEs have strong lipophilicity, so they were easy to accumulate in adipose tissue. Muscle tissue and adipose tissue from different parts of the body were analyzed. Interestingly, the PBDE concentrations were lower in the hind leg muscle than the chest muscle and the PBDE concentrations were lower in the tail fat than the abdominal fat. This indicates that the distance of blood delivery from the core organ affected the PBDE concentration in the tissues.

### 3.2. PBDE Congener Patterns in Different Organs and Tissues

**Figure 2:**
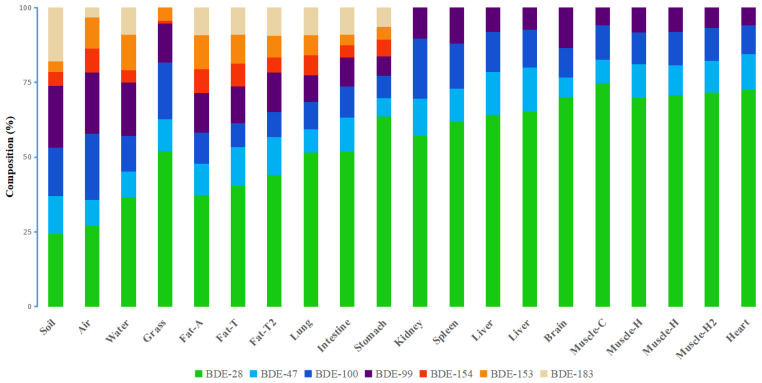
Polybrominated diphenyl ether congener patterns for the different tissues or organs from a sheep raised in a remote grassland area. Notes: Fat-A = abdominal fat, Fat-T = tail fat, Fat-T2 = tail fat of the second sheep, Muscle-C = chest muscle, Muscle-H = hind leg muscle, Muscle-H2 = hind leg muscle of the second sheep.

The PBDE congener patterns in the tissues and organs were not completely similar, as shown in Figure 2. The dominant PBDE congener in all tissues and organs was BDE-28. The more-brominated BDE-154, -153, and -183 were only found in the adipose, lung, small intestine, and stomach samples. The BDE-154, -153, and -183 made a small contribution to the total PBDE concentrations in the soil (26.3%) and grass (5.32%). BDE-183 was not detected in the grass samples. The BDE-154, -153, and -183 contributed 16.4%, 16.6%, and 22.7% of the total PBDE concentrations in the stomach, small intestine, and lung samples, respectively, indicating that sheep take up PBDEs by intaking grass and soil. Blood in the pulmonary circulation system is distributed from the systemic circulation system, but pollutants entering the pulmonary circulation system have not been degraded by the liver in time [36,37]. The pulmonary artery is thinner than the systemic circulation arteries [38]. In the process of pulmonary circulation, blood flows to the capillary network around the alveoli for gas exchange. Therefore, it may be difficult for BDE-153, -154 and -183 to be absorbed by pulmonary capillaries and transported by blood circulation to other tissues and organs. Similarly, BDE-153, -154 and -183 ingested into the stomach were also difficult to enter the blood and transported to other tissues and organs. The more-brominated BDE congeners (BDE-153, -154 and -183) contributed 30.6% and 29.9% of the total PBDE concentrations in the abdominal fat and tail fat samples, respectively. So, more-brominated BDEs (which have high logK_OW_) were more readily stored in adipose tissue, than other tissues and organs. It is interesting to note that more-brominated BDEs were not detected in the kidney, liver, muscle, spleen, heart, and brain samples. This may be because the more-brominated BDEs in the intake organ (stomach) were difficult to transport to the internal organs. Another reason may be the degradation of more-brominated BDEs by liver [39]. The brain is rich in lipids, but the blood–brain barrier prevents more-brominated BDE congeners accumulating in brain tissue. More-brominated BDEs have been found to be prevented from accumulating in the brains of many animals, including chickens, polar bears, and other vertebrates (ducks, fish, frogs, and mice) [6,40,41]. The blood–heart barrier is thought to prevent some substances from entering the heart [42,43]. More-brominated BDEs were not detected in the sheep heart, possibly because they could not cross the blood–heart barrier. The distribution of PBDEs in sheep tissues will be controlled not only by the different functions of tissues and organs but also by the differences in physicochemical characteristics of the different PBDE congeners.

**Figure 3:**
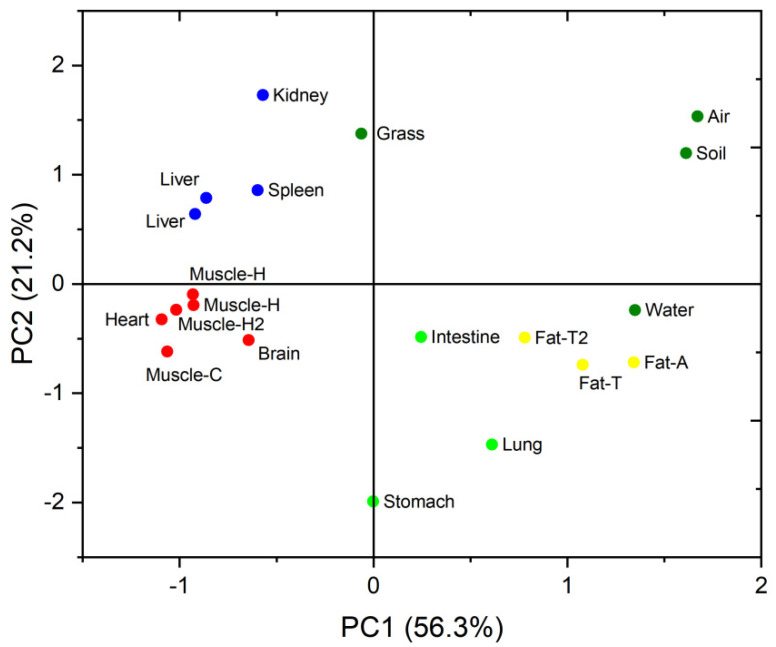
Two-dimensional principal component analysis score plot for the polybrominated diphenyl ether concentrations in the sheep tissue samples.

Principal component analysis (PCA) was performed to identify similarities and differences between the PBDE congener patterns of the different samples. The PBDE concentrations were converted to percent contributions to the sums of the PBDE concentrations before PCA was performed. Principal components (PCs) with eigenvalues > 1 were used. Two PCs explained 77.5% of the variance in the data, as shown in Figure 3. The brain, heart, kidney, liver, muscle, and spleen samples were on the left-hand side of the PCA score plot, the adipose tissue samples were on the middle of the right of the plot, the lung, small intestine, and stomach samples were scattered through the middle and lower part of the plot, and the environmental samples were located on the upper right part of the plot. The respiratory organ (lung) and digestive organ (stomach and small intestine) were in contact with air, water, soil particles, and food. Therefore, the distribution of PBDEs in these organs may be affected by external exposure and internal exposure. The distribution of PBDEs in adipose tissue was inconsistent with that in other organs. This indicates that the ability of adipose tissue to accumulate PBDEs was different from other tissues or organs. The adipose tissue readily accumulated more-brominated BDEs but the other tissues and organs mainly accumulated less-brominated BDEs. Voorspoels et al. determined PBDE concentrations in various tissues from birds and also found different PBDE bioaccumulation patterns in adipose tissue from other tissues and organs [8]. Liu et al. suggested that most tissues and organs of ducks can quickly reach equilibrium or stable state after eating PBDEs, but adipose tissue can be continuously enriched [44]. Zheng suggested that the PBDE distribution pattern in chickens was different between adipose tissue and other tissues and tissues [31].

### 3.3. Selective Bioaccumulation of PBDE Isomers in the Sheep Tissues and Organs

**Figure 4:**
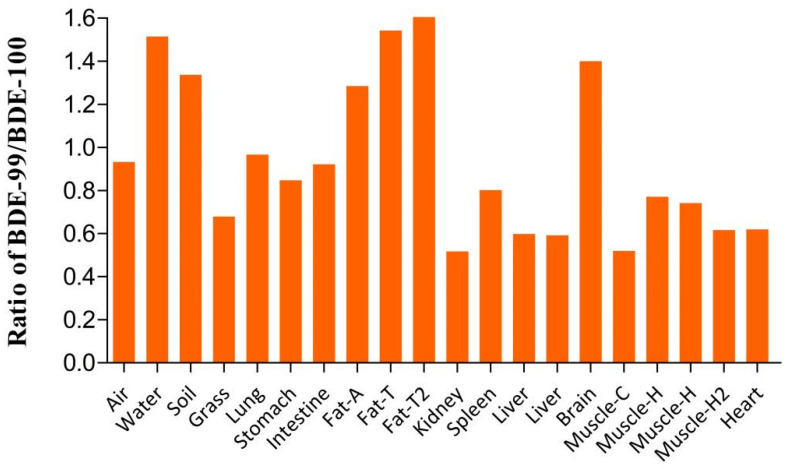
BDE-99/BDE-100 ratios in the environmental media and sheep tissue samples.

The isomers BDE-99 and -100 have very similar physical and chemical properties. The BDE-99/BDE-100 ratio can be used to indicate the selectivity with which different PBDE isomers are distributed to different organs and tissues in an organism [45]. The BDE-99/BDE-100 ratios were lower for the grass samples than the air and soil samples (Figure 4). This indicated that PBDE isomers are selectively enriched in grass when they are taken up from other environmental media [28]. The BDE-99/BDE-100 ratios for the small intestine and stomach samples were between the BDE-99/BDE-100 ratios for the grass and soil samples. The BDE-99/BDE-100 ratio for the lung sample was between the BDE-99/BDE-100 ratios for the air and soil samples. This indicated that PBDEs taken up from both food and other environmental media were selectively enriched in the lung, small intestine, and stomach. PBDE isomers were also selectively enriched in other tissues and organs. The ratio of BDE-99/100 for central organs and tissues (kidney, spleen, liver, heart, and muscle) was less than 1. This means that these organs and tissues can selectively enrich BDE-100. The BDE-99/BDE-100 ratio for the adipose tissue and brain were greater than 1. This indicated that BDE-99 was selectively enriched in the adipose tissue and brain.

Nomiyama et al. found BDE-99/BDE-100 ratios >1 for the brains of stray dogs in Japan but BDE-99/BDE-100 ratios < 1 for the livers [33]. Yang et al. found BDE-99/BDE-100 ratios > 1 for the brains of hens and ducks, indicating that the blood–brain barrier can be crossed more readily by BDE-99 than BDE-100 [46]. They suggested that this may be because steric hindrance affects the crossing of PBDE isomers through the blood–brain barrier. We attempted to improve the understanding of the selective bioaccumulation of PBDE isomers in certain tissues by assessing the molecular characteristics of different PBDE congeners.

### 3.4. Abilities of PBDEs to Bind to Ovine Serum Albumin (OSA) and Cytochrome P450 Enzyme (CYP3A24)

**Figure 5:**
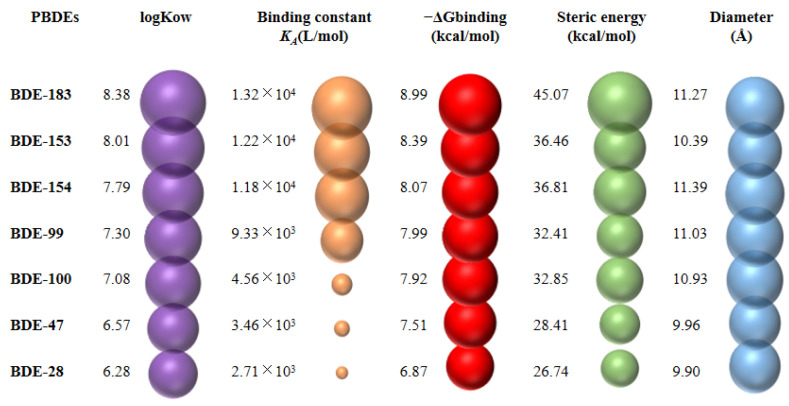
Binding abilities and binding free energies for interactions between PBDE congeners and ovine serum albumin (OSA) and steric hindrance energies for PBDE congeners (bubble width represents data size).

The fluorescence spectra for mixtures of PBDEs and OSA at room temperature (27 °C) were acquired and are shown in Figure S1 and Table S1 in Supplementary Materials. Molecular docking was used to visually show the interaction between PBDEs and OSA, and explore the relationship between the binding energy of molecular docking and the binding constant obtained by fluorescence spectra. A diagram showing the binding constants (*K_A_*) between PBDEs and OSA and the molecular docking binding energy (−ΔG_binding_) between PBDEs and CYP3A24 is shown in Figure 5. The *K_A_* between PBDEs and OSA showed an increasing order of BDE-28 < BDE-47 < BDE-100 < BDE-99 < BDE-154 < BDE-153 < BDE-183. It is worth noting that the order of −ΔG_binding_ values is consistent with *K_A_* values. A large −ΔG_binding_ value means strong affinity [27]. This indicates that the molecular docking simulation results are consistent with the experimental results of fluorescence spectra. The higher the *K_A_* (or −ΔG_binding_), the stronger the binding of PBDEs to OSA, and the greater the possibility of PBDEs being transported to the liver. The high *K_A_* (or −ΔG_binding_) of BDE-153, -154, and -183 mean that these congeners can bind more strongly than other PBDE congeners with OSA and therefore be transported more readily than other PBDE congeners to the liver. This can partly explain why these more-brominated BDEs were not detected in the kidney, liver, muscle, and spleen samples. Transepithelial transport of PBDEs mainly occurs through passive diffusion [47]. The more-brominated BDE-153, -154, and -183 were not detected in the brain and heart samples, possibly because the blood–brain and blood–heart barriers prevented these PBDE congeners entering the brain and heart, respectively.

**Figure 6:**
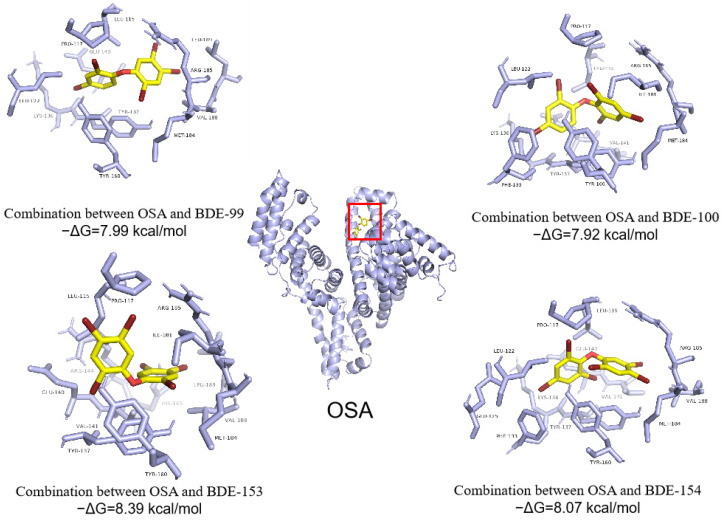
Molecular docking images for interactions between PBDE congeners and ovine serum albumin (OSA) at 298.15 K and the amino acid residues in OSA that interact with the PBDE congeners over the range 4 Å.

Molecular docking of the interaction between PBDE isomers (BDE-99 and BDE-100, BDE-153 and BDE-154) and OSA was used to explain the transport of PBDE isomers by OSA to the liver (Figure 6). The −ΔG_binding_ for interactions between BDE-99 (which contains bromine atoms at two ortho positions, two para positions, and one meta position) and OSA was 7.99 kcal/mol, which was higher than the −ΔG_binding_ (7.92 kcal/mol) for interactions between BDE-100 (which contains bromine atoms at three ortho positions and two para positions) and OSA. The −ΔG_binding_ for interactions between BDE-153 (which contains bromine atoms at two ortho positions, two para positions, and two meta positions) and OSA was 8.39 kcal/mol, which was higher than the −ΔG_binding_ (8.07 kcal/mol) for interactions between BDE-154 (which contains bromine atoms at three ortho positions, two para positions, and one meta position) and OSA. The PBDE isomers with bromine atoms at two ortho positions (BDE-99 and -153) will more readily interact with OSA than the PBDE isomers with three bromine atoms at ortho positions (BDE-100 and -154).

**Figure 7:**
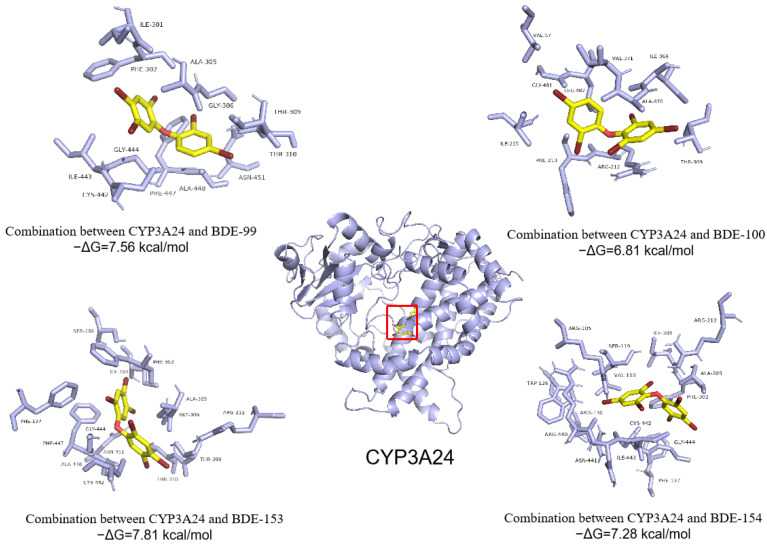
Molecular docking images for interactions between PBDE congeners and the Cytochrome P450 enzyme (CYP3A24) at 298.15 K and the amino acid residues in CYP3A24 that interact with the PBDE congeners over the range 4 Å.

The molecular docking of the interaction between PBDE isomers and CYP3A24 enzyme was used to explain the metabolism of PBDE isomers by the CYP3A24 enzyme in the liver (Figure 7). Interestingly, the −ΔG_binding_ values for interactions between PBDE isomers with different bromine substitution positions and the metabolic enzyme CYP3A24 were consistent with the results between PBDE isomers and OSA. The −ΔG_binding_ for interactions between BDE-99 and CYP3A24 was 7.56 kcal/mol, which was higher than the −ΔG_binding_ (6.81 kcal/mol) for interactions between BDE-100 and CYP3A24. The −ΔG_binding_ for interactions between BDE-153 and CYP3A24 was 7.81 kcal/mol, which was higher than the −ΔG_binding_ (7.28 kcal/mol) for interactions between BDE-154 and CYP3A24. Therefore, the PBDE isomers with bromine atoms at two ortho positions (BDE-99 and -153) will more easier to be transported by OSA to the liver and metabolized by CYP3A24 enzyme in the liver than the PBDE isomers with three bromine atoms at ortho positions (BDE-100 and -154).

Molecular docking between PBDE isomers and OSA showed that PBDEs bound to the OSA hydrophobic cavity, which was composed of amino acid residues GLU, LEU, LYS, ARG, TRY, UAL, PRO, ILE, and MET (Figure 6). PBDE isomers bound to the CYP3A24 hydrophobic cavity, which was composed of amino acid residues ILE, PHE, ALA, and GLY (Figure 7). This indicates that the hydrophobic force plays an important role in the formation of the complex. Although the PBDE isomers were all located at the same position of OSA (or CYP3A24), their optimal conformations in the cavity were different. In addition, the amino acid residues around the cavity functional region were also different. Therefore, different interaction forces, including intermolecular hydrophobic force, van der Waals force, and electrostatic force, comprehensively led to different binding forces between PBDE isomers and OSA or CYP3A24. In brief, the distribution of PBDEs in central organs was mainly affected by OSA transport and CYP3A24 enzyme metabolism. However, there are less OSA and CYP enzymes in adipose tissue and brain, so the distribution of PBDEs in adipose tissue and brain was mainly affected by the K_OW_ of PBDEs.

## 4. Conclusions

The levels and distribution of PBDEs in tissues and organs of grassland sheep were different. The sums of the BDE-28, -47, -99, -100, -153, -154, and -183 concentrations in the sheep tissue and organ ranged from 33.4 to 167 pg/g dw (1.68 × 10^2^–1.84 × 10^3^ pg/g lw). The distribution of PBDEs in sheep organs and tissues will be controlled not only by the fuctions of different organs and tissues but also by the characteristics of the different PBDE congeners. The adipose tissue readily accumulated more-brominated BDEs (BDE-154, -153, and -183) but the other tissues and organs mainly accumulated less-brominated BDEs. The distribution of PBDEs in central organs was mainly affected by OSA transport and CYP3A24 enzyme metabolism. The distributions of PBDEs in adipose tissue and brain were mainly affected by the K_OW_ of PBDEs. In this study, the distribution of PBDEs in various tissues and organs of sheep was systematically investigated for the first time, and the transport and metabolism of PBDEs in sheep were explained by fluorescence spectra and molecular docking. This allows us to better understand the bioaccumulation mechanism of PBDEs in mammals. Due to respect for life, only a limited number of sheep were studied in this study, which is the limitation of this study. In addition, more research should be carried out on animals of different ages and different polluted areas.

## Data Availability

Data is contained within the article or supplementary material.

## Supplementary Materials

The following supporting information can be downloaded at: https://www.mdpi.com/article/10.3390/ijerph191912170/s1, Figure S1: Fluorescence spectra of the interaction between PBDE and ovine serum albumin (OSA) at 300 K, λ_ex_ = 280 nm. a−e concentrations were 0, 2 × 10^−7^, 4 × 10^−7^, 6 × 10^−7^, 8 × 10^−7^, and 10 × 10^−7^ mol/L; Table S1: Binding constants and binding sites of PBDE and OSA.
