# Supplementary material for: A Pilot Study on the Concentration, Distribution and Bioaccumulation of Polybrominated Diphenyl Ethers (PBDEs) in Tissues and Organs of Grassland Sheep
Source: Int J Environ Res Public Health. 2022 Sep 26;19(19):12170. doi: 10.3390/ijerph191912170 (PMC9566259; doi:10.3390/ijerph191912170)
Supplement: Supplementary file 1 [file ijerph-19-12170-s001.zip › ijerph-1895111-supplementary.pdf]

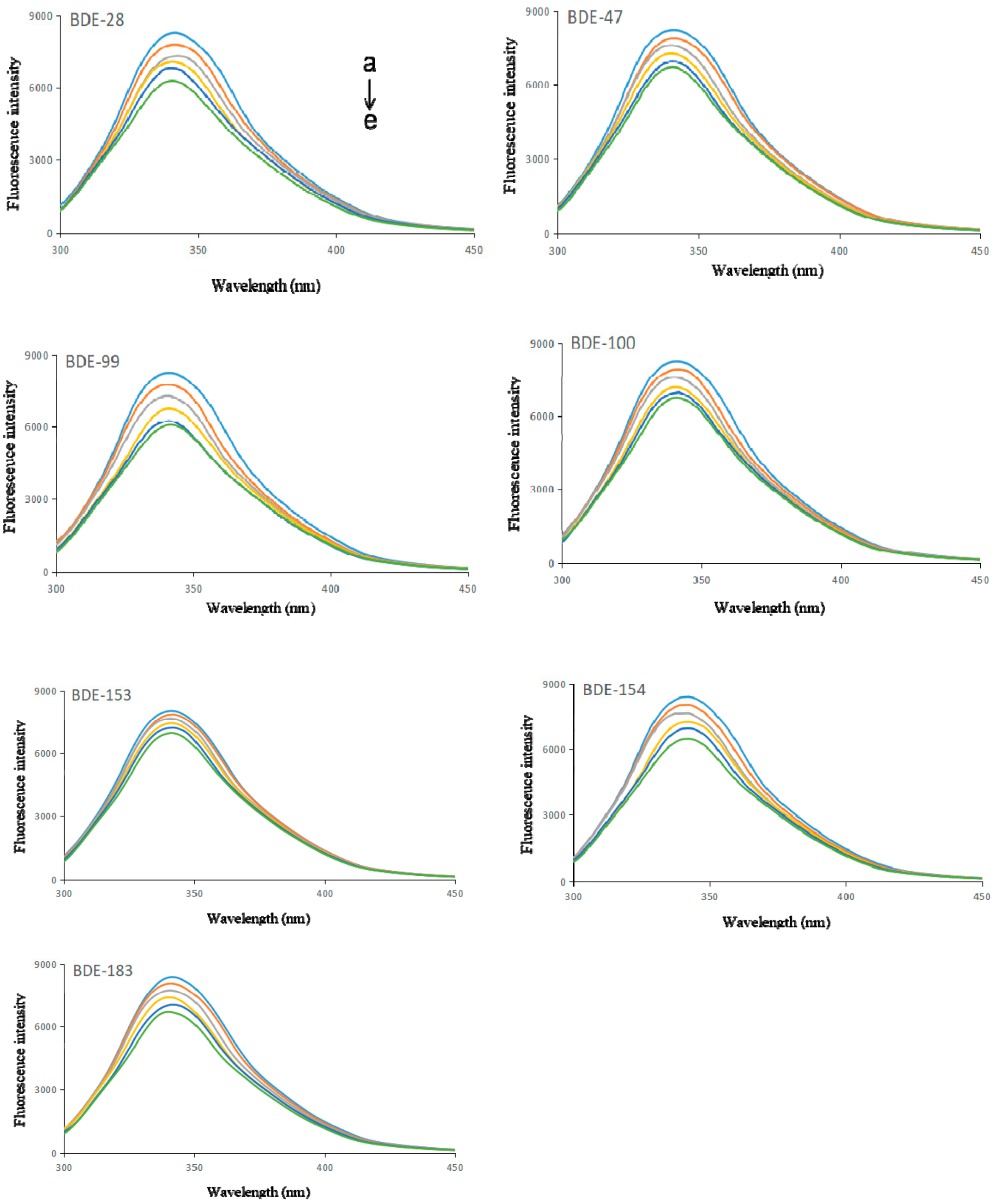

Figure S1. Fluorescence spectra of the interaction between PBDE and ovine serum albumin (OSA) at 300 K, λ_{ex} = 280 nm. a–e concentrations were 0, 2×10^{-7}, 4×10^{-7}, 6×10^{-7}, 8×10^{-7}, and 10×10^{-7} mol/L.

Table S1. Binding constants and binding sites of PBDE and OSA

PBDE	K_A (L/mol)	n	R^2	ΔH (cal/mol)	ΔS (cal/mol)
BDE-183	1.32×10^4	1.1701	0.9992	−10.14	34.25
BDE-153	1.22×10^4	1.2194	0.9992	−9.73	33.97
BDE-154	1.18×10^4	1.1425	0.9972	−9.50	33.84
BDE-99	9.33×10^3	1.0837	0.9923	−9.01	33.00
BDE-100	4.56×10^3	1.0632	0.9908	−8.84	29.42
BDE-47	3.46×10^3	1.0330	0.9994	−8.59	28.53
BDE-28	2.70×10^3	0.9793	0.9916	−8.14	22.10
